# Transition readiness of youth with co‐occurring chronic health and mental health conditions: A mixed methods study

**DOI:** 10.1111/hex.13821

**Published:** 2023-07-14

**Authors:** Brooke Allemang, Susan Samuel, Katelyn Greer, Keighley Schofield, Karina Pintson, Megan Patton, Marcela Farias, Kathleen C. Sitter, Scott B. Patten, Andrew S. Mackie, Gina Dimitropoulos

**Affiliations:** ^1^ Department of Social Work University of Calgary Calgary Alberta Canada; ^2^ Department of Pediatrics, Cumming School of Medicine University of Calgary Calgary Alberta Canada; ^3^ Department of Psychiatry, Mathison Centre for Mental Health Research and Education University of Calgary Calgary Alberta Canada; ^4^ Department of Psychiatry, Cumming School of Medicine University of Calgary Calgary Alberta Canada; ^5^ Department of Pediatrics Stollery Children's Hospital Edmonton Alberta Canada

**Keywords:** mixed methods, patient‐oriented research, transition to adult care, youth mental health

## Abstract

**Background:**

A large proportion of youth with chronic conditions have mental health comorbidities. However, the effect of these comorbidities on paediatric–adult transition readiness, and the relevance of widely used tools for measuring transition readiness, are unknown.

**Objective:**

The objectives of this study were to describe and explore the transition readiness of youth with co‐occurring chronic health and mental health conditions using a combination of quantitative data obtained from participants completing the Transition Readiness Assessment Questionnaire (TRAQ) and qualitative data.

**Design and Participants:**

A three‐phase sequential explanatory mixed methods design was employed, with the qualitative strand taking priority. First, the TRAQ scores (range 1–5) of youth with co‐occurring conditions (*n* = 61) enroled in a multisite randomized controlled trial were measured, followed by qualitative interviews with a sample of youth (*n* = 9) to explain the quantitative results. Results from both strands were then integrated, yielding comprehensive insights.

**Results:**

Median TRAQ scores ranged from 2.86 on the appointment keeping subscale to 5.00 on the talking with providers subscale. The qualitative results uncovered the complexities faced by this group concerning the impact of a mental health comorbidity on transition readiness and self‐management skills across TRAQ domains. The integrated findings identified a diverse and highly individualized set of strengths and challenges amongst this group that did not align with overarching patterns as measured by the TRAQ.

**Conclusions:**

This mixed methods study generated novel understandings about how youth with co‐occurring conditions develop competencies related to self‐care, self‐advocacy and self‐management in preparation for paediatric–adult service transitions. Results demonstrated the assessment of transition readiness using a generic scale does not address the nuanced and complex needs of youth with co‐occurring chronic health and mental health conditions. Our findings suggest tailoring transition readiness practices for this group based on youths' own goals, symptoms, coping mechanisms and resources.

**Patient or Public Involvement:**

This study was conducted in collaboration with five young adult research partners (YARP) with lived experience transitioning from paediatric to adult health/mental health services. The YARP's contributions across study phases ensured the perspectives of young people were centred throughout data collection, analysis, interpretation and presentation of findings. All five YARP co‐authored this manuscript.

## 1 INTRODUCTION

The transition from the pediatric to the adult health system is complex for youth with chronic conditions and their families.^1^, ^2^ Emerging evidence suggests the challenges associated with this transition may be compounded by the presence of mental health comorbidities, medical complexity, poverty and other social determinants of health.^3^, ^4^, ^5^ Assessing youths' readiness for transition to adult care to identify gaps in skills and knowledge that would require intervention has been identified as an important component of transition planning in best practice guidelines.^2^, ^6^, ^7^ Studies suggest that youth who demonstrate effective self‐care, self‐advocacy and decision‐making skills transfer more successfully to adult care and have better health outcomes, including higher self‐reported medication adherence.^8^, ^9^, ^10^, ^11^, ^12^ A neglected topic in this literature, however, is the role of mental health comorbidities in transition readiness in youth with chronic conditions.

There are a variety of transition readiness tools available to measure domains related to self‐management, including knowledge and skill mastery, level of preparedness to engage in self‐care tasks, and self‐efficacy.^13^, ^14^ The Transition Readiness Assessment Questionnaire (TRAQ)^15^ has been deemed the best available readiness assessment given it is well‐validated, theory‐informed and disease‐neutral.^14^ While the TRAQ has been studied and validated in youth with physical, developmental and mental health diagnoses,^16^ research examining its applicability for those with co‐occurring chronic health and mental health conditions is limited. Up to 59% of youth with chronic health conditions experience mental health comorbidities;^17^, ^18^ however, the impact of youths' mental health and well‐being on transition readiness as measured by the TRAQ has not been adequately explored. Thus, this study aimed to explore and describe relationships between mental health comorbidities and transition readiness (as assessed by the TRAQ) in youth with chronic health conditions using a combination of quantitative and qualitative data.

## 2 METHODS

### 2.1 Study design

**Figure 1:**
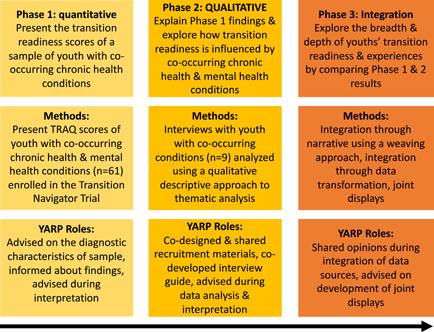
Sequential explanatory mixed methods study design by phase. TRAQ, Transition Readiness Assessment Questionnaire; YARP, young adult research partner.

This research adopted a three‐phase patient‐oriented, sequential explanatory mixed methods design,^19^ with the quantitative arm as the supplemental component (phase 1) and the qualitative arm (phase 2) as the core component (quan → QUAL; see Figure 1).^20^ This qualitatively driven design was selected given the study's overarching objective of exploring and describing the transition readiness of youth with co‐occurring chronic health and mental health conditions.^20^, ^21^ Median TRAQ^15^ subscale scores were gathered and analyzed for youth with co‐occurring chronic health and mental health conditions enroled in a multisite randomized controlled trial (the Transition Navigator Trial [TNT]^22^) in Alberta, Canada. Next, qualitative interviews were conducted with a sample of youth with co‐occurring chronic health and mental health conditions at various stages of the healthcare transition process to elaborate on, enhance, and explain the quantitative findings.^23^ While the methods and results of the quantitative and qualitative phases of this study have been published separately,^3^, ^24^ this article describes and presents novel insights arising from the integration of the two data sources.

The rationale for adopting this mixed methods design was complementarity, wherein two data sources are used in tandem to provide a more comprehensive understanding of the transition readiness of this group than would have been possible with either quantitative or qualitative data alone.^23^ Through combining and jointly interpreting the TRAQ score data and qualitative interviews with youth, this research sought to generate practical recommendations for care providers supporting this population in preparing for health service transitions.^25^ Reporting of this study's findings adheres to the Good Reporting of A Mixed Methods Study framework^26^ (Supporting Information: File 1).

### 2.2 Patient and public involvement

**Figure 2:**
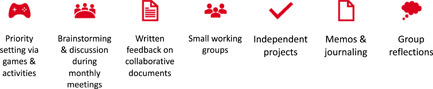
Menu of options for young adult research partner engagement.

This study was conducted in collaboration with five young adults with lived experience in the health and/or mental health systems, using a patient‐oriented research approach.^27^ The experiential knowledge and perspectives of the young adult research partners (YARP) were integrated across study phases to ensure our questions aligned with their priorities, and to generate findings relevant to young people themselves. YARP members were aged 18–30 years, resided in Canada and had lived experience transitioning from pediatric to adult health/mental health services. Further details about the formation, onboarding and processes of YARP engagement have been previously described.^3^, ^24^, ^28^ Several opportunities and possible tasks for YARP members according to the study phase were agreed upon by the YARP in a team discussion. These tasks included collaborating on: (i) the interview guide; (ii) qualitative data interpretation; (iii) preparing presentations/articles and (iv) the knowledge translation strategy (see Figure 1 for YARP roles according to phase). Given YARP members had different hopes of contributing to the project based on their preferences (e.g., oral vs. written input), a menu of options for engagement was developed in partnership with the YARP (see Figure 2) so they had autonomy over their involvement.

**Figure 3:**
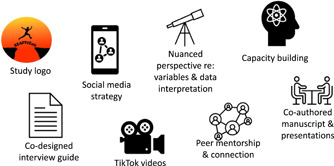
Young adult research partner contributions and outputs.

The YARP met virtually 17 times between March 2021 and September 2022. Their contributions ranged from tangible outputs, including a YARP‐designed study logo and social media strategy, to process‐based outputs, including a nuanced perspective on study variables and data interpretation that was enhanced based on the lived experiences of the members (see Figure 3 for a summary of the YARP's contributions and outputs).

### 2.3 Phase 1: Quantitative data

Phase 1 involved a secondary analysis of data collected for the TNT, a trial designed to assess the effectiveness of a patient navigator for youth with chronic conditions transitioning to adult health care in Alberta.^22^ Though the methods and results of our phase 1 analysis have been published elsewhere,^24^ an overview of information relevant to the mixed methods design follows. Eligible participants for this secondary analysis: (i) were between 16 and 18 years old; (ii) were followed in a chronic disease clinic at one of the three study sites; (iii) identified as having a mental health comorbidity; (iv) resided in Alberta; (v) were expected to transfer to an adult specialist and (vi) had to have completed the TRAQ and baseline demographic forms at study enrolment. Individuals with intellectual/developmental disabilities (IDD), or chronic health/mental health conditions only (but not both), were excluded from the phase 1 cohort given our study's objective of examining transition readiness in youth with co‐occurring chronic health and mental health conditions and the fact that many youth with IDD in our sample did not complete the TRAQ for themselves. While a total of 334 patients were enroled in the TNT, 61 individuals met inclusion criteria for this secondary analysis.

All phase 1 participants completed the TRAQ^15^ at study enrolment. This tool is a 20‐item self‐report questionnaire that assesses transition preparedness, self‐management skills and disease‐related knowledge in youth with chronic conditions.^15^ The TRAQ contains questions related to five subscales: managing medications, appointment keeping, tracking health issues, talking with providers and managing daily activities.^15^ Responses are scored using a Likert‐type scale which ranges from 1 to 5 with higher scores depicting greater readiness for transition.^15^ The tool produces an overall transition readiness score by averaging the scores of all 20 items, and scores for each of the five subscales using the same approach.^15^ In this secondary analysis, the TRAQ subscale scores of youth with co‐occurring chronic health and mental health conditions were described using medians and interquartile ranges (IQRs).

### 2.4 Phase 2: Qualitative data

While the methods associated with phase 2 have been described in a prior publication,^3^ this article presents novel integrated insights specific to participants' perceptions of the TRAQ and feelings of preparedness for adult care across the TRAQ subscales. In phase 2, qualitative interviews with a purposive sample of youth with co‐occurring chronic health and mental health conditions aged 16–20 were conducted to explore the impact of their mental health on transition readiness and self‐management skills (e.g., communicating with providers, managing medications). Participants were selected based on their age, gender and diagnoses, allowing for a range of experiences to be captured. We aimed to recruit between 10 and 15 participants for phase 2 based on prior literature.^29^ However, data collection and analysis co‐occurred, and we ceased recruitment efforts once our research question had been addressed,^30^ resulting in nine participants.

Telephone or videoconference interviews lasting between 45 and 60 min were held with participants using a semistructured guide co‐developed with the YARP.^3^ Given this study adopted a sequential explanatory design, the quantitative results informed the qualitative interview questions, allowing us to build on the phase 1 findings. The narratives of youth were elicited to help provide explanations for the phase 1 median TRAQ subscale scores. Phase 2 participants reviewed the TRAQ in advance of the interviews to consider their own readiness for adult care and how their mental health influenced preparedness to engage in tasks associated with each subscale domain. The interviews were audio recorded, transcribed verbatim and imported into NVivo software^31^ for analysis. An inductive approach to qualitative description^32^ using reflexive thematic analysis^33^ was used to identify relevant codes and resulting themes.

Throughout the analysis, the research team (including the YARP) reviewed interview transcripts, reflected on the meaning of participants' experiences, inductively coded the transcripts and generated initial themes.^33^ The first author and the YARP met monthly to discuss prominent concepts and codes, and illustrative quotes and to share our impressions of the data. The YARP offered feedback on the codes, themes and visuals using various methods, including verbal input in meetings, written comments on a shared document, brainstorming activities using a Google Jamboard, reflexive journal entries/memos and/or by email. Each team member brought their own experiences and perspectives into the analytic process and the senior author was available to resolve conflicts (though this was not required). Through collaborative and reflexive group discussions and using the aforementioned methods, the team developed the resulting themes and subthemes.^33^

### 2.5 Phase 3: Integration

There were two points of integration during this study: during data collection and data analysis. The results from the descriptive analysis of the TRAQ scores in phase 1 guided the types of questions asked during the qualitative interviews in phase 2. Specifically, youth were invited to reflect on which areas of the TRAQ (e.g., managing medications, talking with providers) they felt most/least confident in, allowing for comparison with the median TRAQ subscale scores from phase 1. The second point of integration took place during data analysis at the reporting level.^34^ We integrated the findings through narrative using a weaving approach whereby the quantitative and qualitative findings were described together according to the theme.^34^ The narrative was developed by reflecting on how both data sources could be used in a complementary fashion to contribute to newfound understandings about the ways in which youth with co‐occurring conditions prepare for the transition. The resulting integrated themes were guided by the concepts assessed in each of the five TRAQ subscale domains. Finally, we developed side‐by‐side presentations and joint displays in partnership with the YARP to illustrate the similarities and differences between the two data sources according to the TRAQ domain.^34^, ^35^

## 3 RESULTS

### 3.1 Participants

**Figure 4:**
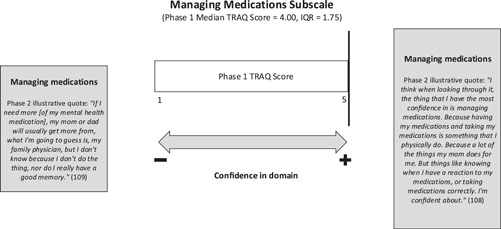
Joint Display of TRAQ managing medications subscale results. IQR, interquartile range; TRAQ, Transition Readiness Assessment Questionnaire.

**Figure 5:**
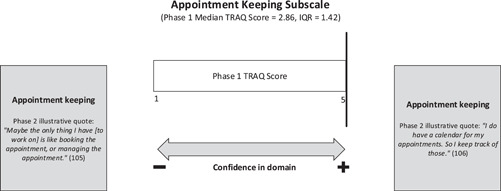
Joint display of TRAQ appointment keeping subscale results. IQR, interquartile range; TRAQ, Transition Readiness Assessment Questionnaire.

**Figure 6:**
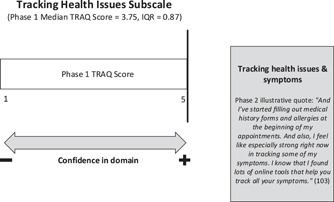
Joint display of TRAQ tracking health issues subscale results. IQR, interquartile range; TRAQ, Transition Readiness Assessment Questionnaire.

**Figure 7:**
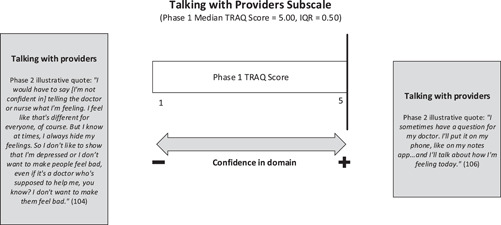
Joint Display of TRAQ talking with providers' subscale results. IQR, interquartile range; TRAQ, Transition Readiness Assessment Questionnaire.

**Figure 8:**
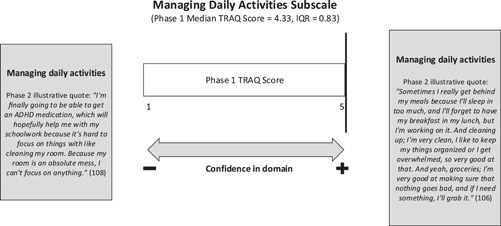
Joint Display of TRAQ managing daily activities subscale results. IQR, interquartile range; TRAQ, Transition Readiness Assessment Questionnaire.

The demographic and clinical characteristics of the quantitative and qualitative samples are available in previous publications.^3^, ^24^ Briefly, our phase 1 sample consisted of 61 individuals with co‐occurring health and mental health conditions. The mean age was 16.9 years, and the majority of participants were cisgender women (68%), White (72%) and Canadian‐born (95%). In phase 1, the most commonly reported chronic health conditions were rheumatological (36%), neurological (16%), endocrine (13%) and gastrointestinal/liver (13%) and the most commonly reported mental health comorbidities were anxiety (82%), depression (57%) and attention deficit hyperactivity disorder (ADHD; 26%). The median TRAQ subscale scores for the 61 participants in phase 1 are presented in Table 1 and in the five joint displays (Figures 4, 5, 6, 7, 8) below. Nine individuals were interviewed for phase 2; of note, none of these participants were part of the phase 1 cohort. The mean age of our phase 2 sample was 18.3 years, and most participants were cisgender women (56%), White (67%) and Canadian‐born (78%). The most commonly reported chronic health conditions in phase 2 were respiratory (67%), gastrointestinal (22%), genetic (22%) and rheumatological (22%) and the most commonly reported mental health comorbidities were anxiety (89%) and depression (67%).

**Table 1:** Juxtaposed findings from phase 1 (quantitative) and phase 2 (qualitative).

	Phase 1 TRAQ score for youth with co‐occurring diagnoses (*n* = 61)			
TRAQ subscale	Median	IQR	Phase 2 domains related to TRAQ subscale (*n* = 9)	Phase 2 demonstrative quote(s)
Managing medications	4.00	1.75	Confident in managing medications	I've always been in charge of my medications, like not ordering them, but taking them correctly on my own. I've had medications since I was like five so that's always been a thing that I'm used to. (103)
				The thing that I have the most confidence in is managing medications. Because having and taking my medications is something that I physically do. Because a lot of the things my mom does for me. But taking my medications is really upfront, so I feel more confident than that. Like knowing when I have a reaction to my medications, or taking medications correctly, I'm confident about. (108)
			Mental health symptoms impact medication management and efficacy	At first, during my initial stage of being diagnosed [with diabetes], I had kind of given up and lost hope. So at first, I didn't take medication. (105)
				I feel like I always need to be in the yes portion and being like “good”, even though there are some days mentally and just like physically, I cannot like call to fill a prescription for example. Or like some of these you're just like, yep, I cannot do these on a bad day. (102)
				With every new start of medication, there's anxiety that comes with that and that causes more flares so you can never really tell what's working and what's not because the anxiety is throwing it into a flare. So I feel like my mental health has always made it difficult to try and control my lupus. (103)
				When I used to get like really anxious, I would forget to bring my puffer, and then I would just be hyperventilating to the point where I would get really lightheaded. (106)
				When depression gets so intense, you don't really care about yourself and you don't care to look after yourself. And so in those moments…I would purposefully not take my medication…because I knew that that was just going to make me feel worse. (101)
			Medications cause mental health side effects	My doctors didn't talk to me about the mental health effects that some of my medications actually resulted in, and some of the withdrawal, addiction, and mental health issues that come with some of these stronger steroids and drugs, which have always been an issue for me. (103)
				When it comes to physical health, the way my conditions are linked. If my medication is off for my hypothyroidism, then I'll feel more sluggish and tired. And that will usually throw me into depression just because I feel like I'm slacking. (107)
Appointment keeping	2.86	1.42	Mental health symptoms affect appointment keeping and attendance	I think I definitely could be more ready. If I didn't have the lack of motivation to step up and file my taxes, or write down when my appointments are, or keep track of all this paperwork and stuff like that… I do feel like that impacts [my transition readiness], and I don't quite know what to do about it. So I just kind of get in this position where I feel stuck. And then I just kind of don't do things when I'm supposed to do the things, you know? And if I'm in an appointment and someone will ask me, when do you have an appointment? Or, have you been to this appointment? I just don't know. (108)
				I think even going to go get medication or going to go see my doctor's appointments it was made a lot more difficult with the anxiety. And again, the anxiety piece made it very difficult for me to get on public transit and that actual realistic aspect of getting to my appointments was already made a lot more difficult with my social anxiety. (101)
				I feel like I was a little bit scared [on my way to my first adult appointment]. I got uncomfortable, and my anxiety really added pressure, and I started to worry lots about what was going to happen, if I was going to be late because I couldn't find out where I was going. I sometimes I get really disoriented, especially like if I'm on a train, or I've been running to catch my train, and I forget what I'm doing. I don't know why I can't even figure out where I'm going and run at the same time without getting overwhelmed, so I was worried that was going to happen, and then I would be late for my appointment. And I know my neurologist is busy, so I didn't want to do that. (106)
				Remembering appointments [is challenging for me]. I don't have my driver's license quite yet, so I can't drive myself to appointments either. I've never been to an appointment without my mom before, except for the one for my ADHD. Yeah, I'm honestly quite nervous and I'm not sure how ready I feel, but I just kind of trying to go with it. (108)
			Lack of knowledge of health insurance	I'm not confident in the health insurance and the budgeting household expenses, because the money and budgeting household expenses is something my dad does, and I don't know anything about my health insurance. (109)
				I was very anxious around the idea of health insurance during my transition into adult care especially because my parents work for themselves and so they have their own independent insurance. Thankfully, I'm covered under their insurance but it was definitely a scary moment of thinking, oh my gosh, what am I going to have to do if I can't get my medications covered? (101)
Tracking health issues	3.75	0.87	Use of tools to promote organization and confidence in tracking symptoms	I do have a calendar for my appointments. So I keep track of those. (106)
				I feel especially strong right now in tracking some of my symptoms. I found lots of online tools that help you track all your symptoms and all your medications which has been super helpful. And even the My Health app that Alberta has now, I found it to be really helpful because you can have notes and it also has all your medications already down for you. (103)
				I made this book [my health journal] where I stated my name, my date of birth, my health conditions, my different doctors. I have my family doctor, eye specialist, ear specialist, and someone to do my MRIs. So I just wrote all of that down in here. I have an idea of what my typical stats are. So I wrote down usually I'm at 97.6 Fahrenheit for my temperature. Usually, I'm around 98 percent for my oxygen. My BPM is usually quite calm. It's usually high fifties, low sixties. And my blood pressure is usually quite well. I use everything like that so that I am able to answer the questions. And if I'm incapacitated, then [health care providers] can look at that book. (107)
				I think writing the important things down, for me specifically, would be super helpful. Either I write things down, or [health care providers] write things down. Like they give notes throughout the session. And then I get them at the end so I can keep track of things just because of my memory issues. I do feel like that would be a specific thing that would help me. And I imagine it would help other people who have memory issues, or ADHD, who are forgetful so they can have those notes with them. (108)
Talking with providers	5.00	0.50	Confident in communicating with healthcare providers due to interactions with a variety of clinicians	For me, I think I'm kind of ready because I've interacted with different providers and I think I've built up courage. So explaining how I'm feeling, how the medications are affecting me, if I have any problems taking the medications. These kinds of questions, I'm ready to answer. (105)
				I think even the idea of having multiple chronic illnesses just meant more exposure to having to explain myself to multiple different providers and providers that I hadn't been with throughout my diagnosis process and so they didn't know my health history. It definitely exposed me to the experience of having to re‐articulate myself and to be able to vocalize my pain and my health history. Just being used to the process of going to doctor's appointments more often and meeting with a diverse team of healthcare providers definitely helped [with my readiness]. I think the frequency in which I saw healthcare providers impacted how comfortable I felt because I think with most things, practice makes perfect. (101)
			Mental health symptoms create challenges with communication	I would have to say as well, [I lack confidence in] telling the doctor or nurse what I'm feeling. I feel like that's different for everyone, of course. But I know at times, I always hide my feelings. So I don't like to show that I'm depressed or I don't want to make people feel bad, even if it's a doctor who's supposed to help me, you know? I don't want to make them feel bad. (104)
				It's kind of nerve racking for whatever reason to make the calls to your doctors or when I'm in the appointments, I get nervous and look to my mom even though I know what's happening with my some of my health stuff, you just get nervous to talk to your doctor about some of the things. (103)
				It was a little bit weird going [to my first adult appointment] alone, and I felt like, I didn't know exactly if I was doing it correctly. It was nerve‐racking because the doctor would ask questions and then I'd answer, then I wasn't sure if that's what they wanted or if I was doing it wrong. So it was a little bit harder to deal with. (106)
				I'm still stressed [about transitioning to adult care] because I'm scared of how to interact with people and talk to doctors about unusual changes in my health. While I haven't been officially diagnosed with social anxiety, I myself believe I have it. Because if I say something wrong to someone, I feel like they're going to hate me for the rest of my life; even if it's something nice, they'll take it some way wrong and they'll hate me. So I'm really afraid of having to interact with people instead of someone doing it for me. (109)
				I get anxious with talking to people in person, or at doctor's appointments. Because sometimes when I'm anxious I'll just go just completely silent. I'm like, I don't know what to say. Am I supposed to say something? Oh no. And then I just spiral. (108)
				Calling the doctor's office, I still have that fear…I can't do it without my mom. (105)
Managing daily activities	4.33	0.83	Challenges completing activities of daily living when mental health symptoms are present	I'm finally going to be able to get an ADHD medication, which will hopefully help me with my schoolwork because it's hard to focus on things with like cleaning my room. Because my room is an absolute mess, I can't focus on anything. (108)
				My meal planning sometimes is really bad. Sometimes I get behind with my meals because I'll sleep too much, and I'll forget to have my breakfast in my lunch, but I'm working on it, working on it. (106)
				It's difficult to sometimes have the motivation to do the important things, or even just my hobbies in general. I'm super passionate about drawing and art. And I have been passionate about it for pretty much my whole life. But sometimes I just can't bring myself to draw… (108)

Abbreviations: ADHD, attention deficit hyperactivity disorder; IQR, interquartile range; TRAQ, Transition Readiness Assessment Questionnaire.

### 3.2 Mixed methods insights

There was some discordance^34^ between the phase 1 and phase 2 results regarding the impact of a mental health comorbidity on transition readiness. Several youth described developing transition readiness as a result of overcoming mental health challenges, engaging with a series of care providers (including mental health providers) and practicing self‐management skills in therapy which aligns with our phase 1 findings. However, the qualitative interviews also revealed many youth with mental health comorbidities found their mental health symptoms impeded their ability to communicate with care providers, take ownership of their care and independently navigate a new system. The two data sources used in this study, which reveal at times conflicting results, underscore the complexity of developing transition readiness while living with co‐occurring chronic health and mental health conditions. The integrated results according to the TRAQ subscale are presented below and juxtaposed findings from phases 1 and 2, including demonstrative quotes, are illustrated in Table 1.

#### 3.2.1 Managing medications

Many phase 2 participants indicated feeling confident in managing their medications, whether for their chronic health condition, mental health condition or both, which aligns with the high phase 1 median TRAQ subscale score of 4.00 (see Figure 4). During the qualitative interviews, youth described using adaptive strategies including apps and symptom trackers to assist them in managing their medications. Participants described high levels of confidence in refilling and taking their prescriptions independently because most had been on their medications since childhood. Administering their own medications appeared to be one of the first tasks youth took responsibility for in preparation for transition due to the complexity of their regimens (e.g., steroids and anti‐depressants). While the diversity of experience reflected by the TRAQ subscale score for medication management is identified in the IQR associated with the median score (IQR = 1.75), the phase 2 interviews illuminate some specific and unique challenges faced by individuals with co‐occurring chronic health and mental health conditions in this area.

First, participants described how certain mental health symptoms (e.g., low mood, lack of motivation, inability to focus) affected their capacity to fill prescriptions and independently adhere to medication regimens. Next, two youths described the negative effects of their medications on their mental health, and the specific side effects they observed, including nightmares, withdrawal and depression resulting in the exacerbation of existing mental health symptoms. In addition, several of the youth were prescribed medications (e.g., antidepressants, antianxiety medications) for the management of their mental health, indicating a daily treatment regimen consisting of multiple medications. Taken together, these points reveal the active steps taken by this group to take ownership of medication management in preparation for adult care, while illuminating the complexities they experience regarding treatment regimens and side effects.

#### 3.2.2 Appointment keeping

The median TRAQ appointment keeping subscale score of 2.86 represented the lowest score of all five subscales in the phase 1 sample. The phase 2 results are in alignment with this finding and help to explain why individuals with co‐occurring chronic health and mental health conditions may exhibit lower readiness in this domain. The majority of phase 2 participants expressed the impact of their mental health symptoms, namely anxiety, disorientation and ‘feeling stuck’, on their appointment attendance and appointment keeping capacities (see Figure 5). The examples and experiences of the youth interviewed bring voice to the ways that tracking, remembering and physically getting to and from appointments can be complicated by the presence of a mental health comorbidity.

One youth, for instance, described a lack of motivation to write down appointments and develop a routine for tracking clinic visits as a result of her depression. Taking on this responsibility for the first time in the adult healthcare system without the support of her parents, while simultaneously coping with mental health challenges, complicated the process of tracking and scheduling appointments independently. Another phase 2 participant described the commonly held sentiment of being ‘a little bit scared’ on her way to her first adult neurology clinic appointment. She then elaborated on how her anxiety exacerbated these symptoms and created feelings of pressure regarding the transition to a new provider. This resulted in the youth getting disoriented and overwhelmed as she navigated public transit, adding to the pre‐existing fears associated with attending an appointment independently.

The only TRAQ question which addresses the concept of getting to and from appointments as youth prepare to take ownership over this domain is ‘Do you arrange for your ride to medical appointments?’.^15^ The interview results illuminate the series of challenges experienced by youth with co‐occurring diagnoses regarding appointment attendance, including social anxiety while on public transit, that would not have been apparent based on a score of 1–5 on this subscale.

#### 3.2.3 Tracking health issues

Keeping track of symptoms, completing medical history forms and maintaining a calendar for medical appointments were all skills endorsed by the phase 2 participants in this study. In fact, many phase 2 participants described confidence in their capacities related to the TRAQ ‘tracking health issues’ domain. Furthermore, none of the phase 2 participants outlined this as an area of weakness related to their transition readiness (see Figure 6). The phase 1 median tracking health issues subscale score of 3.75 indicates some discrepancies between the results from phase 2, wherein youth expressed confidence in this domain. The experiences and strategies highlighted by the sample of phase 2 youth could help clarify and account for this finding.

To keep track of the variety of symptoms youth were experiencing related to their chronic health and/or mental health conditions, many Phase 2 participants described using organizational tools, including ‘personal health journals’, ‘the MyHealth app’, ‘online tools for medication and symptom tracking’ and ‘calendars for appointment keeping’. These strategies may be particularly useful for individuals with multiple diagnoses; indeed, all nine interview participants endorsed having at least two (and up to eight) diagnoses. The presence of many chronic conditions necessitates the value of organizational tools to manage symptoms, medications and appointments, especially as youth begin taking ownership of their care in preparation for transition to adult services. It is worth noting that all but one interview participant endorsed being diagnosed with anxiety. The slightly higher proportion of individuals with anxiety in phase 2 compared to those in phase 1 could help explain why there is greater confidence in tracking health issues than would be expected based on the phase 1 results. It is also possible that youth in phase 2 had more manageable levels of anxiety and/or developed more adaptive coping strategies for tracking their health issues, specifically, than those in phase 1.

The combination of results from phases 1 and 2 regarding tracking health issues indicates that factors including the number of comorbidities, complexity and rarity of one's diagnosis(es), and the severity of mental health symptoms influence the ways in which youth track their symptoms and their comfort level in describing their medical history to providers. Appreciating these multifactorial implications on transition readiness would not have been possible looking at the TRAQ tracking health issues subscale scores in isolation.

#### 3.2.4 Talking with providers

The phase 1 results indicated that youth were more confident in talking to providers than any other subscale, with a median score of 5.00 (the maximum possible score for a subscale). As such, there were some unexpected findings arising from the qualitative interviews which counter this level of confidence in communicating with providers.

Some youth in phase 2 expressed feeling very comfortable talking with clinicians, asking questions and describing their symptoms (see Figure 7). These results align closely with the phase 1 findings regarding a high level of confidence for youth with co‐occurring diagnoses in this domain. There were a number of examples provided by interview participants to elucidate reasons for this. For instance, ‘having multiple chronic illnesses’ and ‘being exposed to a variety of providers’ helped the youth ‘build up courage’ to ‘vocalize [their] pain and health history’. As aptly shared by one participant, ‘practice [communicating with health care providers] makes perfect’. Some youth explained how they learned to advocate for their mental health needs which enhanced their comfort in talking with providers. Others felt their ability to be ‘introspective’ regarding their mental health allowed them to articulate their feelings with confidence in clinical encounters.

An unexpected finding arising during the integration of data sources specific to this domain related to the ways in which mental health comorbidities made communicating with providers more challenging. In fact, several youths interviewed expressed a lack of confidence in communicating with their care teams, primarily in relation to their mental health. For some youth, a hesitancy to express their true feelings, particularly related to depression, for fear of disappointing providers contributed to this lack of confidence.

For others, the symptoms of generalized and/or social anxiety made talking with clinicians without caregivers present for the first time stressful. Some youth described the incapacitating effects of anxiety on communicating with care providers. Fears about not having the ‘correct’ answers, not sharing enough information or not asking the right questions during appointments with new adult care providers were exacerbated by pre‐existing anxiety for many youths. They explained the pressure they felt to perform in adult care and how, at times, this pressure led to them shutting down and ‘going completely silent’ so as not to say the wrong thing.

Given the incongruence between the phase 1 and 2 results in this domain, it is possible the two TRAQ questions focused on communication are not sufficiently capturing this phenomenon for youth with co‐occurring diagnoses. While phase 1 participants expressed confidence via their TRAQ subscale scores, youths' experiences indicate the importance of exploring whether youth feel their opinions are valued by providers and their comfort in advocating for their needs and asking questions above and beyond the TRAQ subscale questions.

#### 3.2.5 Managing daily activities

There appeared to be a lack of congruence between the phase 1 median TRAQ managing daily activities score of 4.33 and the narratives of the youth shared in phase 2. The relatively high TRAQ subscale score indicates youth with co‐occurring chronic health and mental health conditions in phase 1 were confident managers of their daily activities, including preparing meals, doing household chores, and using neighbourhood services. While some of the youth interviewed in phase 2 described living independently and being responsible for ‘cleaning’, ‘keeping things organized’ and ‘grabbing groceries’ (see Figure 8), the managing daily activities subscale appeared to oversimplify these concepts for this sample of youth.

Despite the high TRAQ phase 1 subscale score, a number of phase 2 participants shared how their mental health symptoms affected their ability to perform activities of daily living. They described their mental health as ebbing and flowing so that during periods when their mood was low or they were inconsistent with their medications, these activities became challenging to manage independently. Examples were provided of the impact of youths' mental health symptoms on managing daily activities, including lacking the motivation to participate in hobbies they were passionate about (e.g., drawing), difficulty focusing long enough to clean their rooms or complete schoolwork due to ADHD, and challenges with meal planning and preparation due to low mood and extended sleep (see Table 1 for illustrative quotes).

## 4 DISCUSSION

This patient‐oriented, mixed methods study aimed to understand the transition readiness of youth with co‐occurring chronic health and mental health conditions utilizing a combination of quantitative and qualitative data. The phase 1 results, which indicate relatively high TRAQ subscale scores, underscore the role of resilience within this population, whereas phase 2 highlights the criticality of understanding each youth's individual experiences, symptoms, coping strategies and available supports as they relate to transition preparedness. The integrated phase 3 findings necessitate individualized approaches to transition planning which use a combination of readiness assessments, in‐depth conversations and tailored resources given the complexity of this group's needs.

In combining the findings from phases 1 and 2, it became apparent that a transition readiness score using a well‐validated tool is insufficient to capture the breadth of factors contributing to self‐management and preparedness for adult care for youth with co‐occurring health and mental health conditions. It is evident that transition readiness encompasses a variety of skills, capacities, experiences and system‐level factors that may be difficult to address in a single readiness assessment for this group. As has been described by Straus,^13^ assessing transition readiness using a tool like the TRAQ may not adequately capture the evolving and multidimensional nature of this concept and the daily realities of youth preparing for adult care. Thus, there is congruence between this study's findings and Straus' ideas surrounding the challenges associated with measuring transition readiness.^13^ Indeed, our results suggest that the TRAQ may not be sensitive to individual characteristics related to transition readiness for youth with co‐occurring conditions, including the impact of fluctuating mental health symptoms on chronic condition management. In order for clinical decisions and transition plans to be person‐centered, the administration of this tool should be complemented with discussions between youth, families and care providers about self‐management, self‐advocacy and youths' own priorities for transition.

Though the influence of a mental health comorbidity on transition readiness in youth with chronic conditions exiting pediatric care has not been specifically investigated in the literature, research has examined aspects of self‐management amongst individuals with multimorbidity (i.e., the presence of ≥2 chronic conditions).^36^, ^37^ The presence of emotional symptoms (e.g., depression, anxiety) often impairs physical functioning and frequently affects activities of daily living including tasks associated with self‐management in adults with multimorbidity.^36^, ^38^, ^39^ Furthermore, this group is known to experience the compounding nature of physical and emotional symptoms which results in larger negative impacts on their lives and daily routines, echoing the present study's findings.^36^, ^38^, ^39^, ^40^ Our study adds to this body of knowledge by exploring how these concomitant symptoms influence youths' capacities for self‐care during a developmental period in which they are expected to assume greater responsibility for activities of daily living, appointment scheduling and transportation.

Mental health problems fluctuate, thus the need for support with managing daily activities from healthcare providers, family members and/or peers may wax and wane depending on the acuity of mental health symptoms for youth with co‐occurring conditions. The TRAQ, as it is currently worded, does not capture possible temporal variations in readiness based on vacillating symptoms given the questions are prefaced by the following statement: ‘Please check the box that describes your skill level in the following areas that are important for transition to adult health care’.^15^ Without probing qualitatively into the specific interactions between health, mental health, and daily activities, the TRAQ subscale score alone may present an incomplete picture of transition readiness as it relates to this domain for youth with co‐occurring diagnoses. For instance, our qualitative interviews revealed the criticality of clinicians exploring how hobbies, schoolwork or chores are impacted by the manifestation of co‐occurring chronic health and mental health conditions.

While first‐time interactions with adult healthcare providers are anxiety‐provoking for the majority of youth with chronic health conditions,^41^ this study's qualitative data provided greater depth to the TRAQ scores and illustrated the additional barriers experienced by youth with pre‐existing mental health conditions, such as social anxiety in anticipation of appointments. Our integrated insights indicate possible considerations for clinicians supporting youth to develop their confidence across TRAQ domains. These include: practicing communication skills in a scaffolded fashion in pediatric care (i.e., first with caregivers present and then independently once youth develop confidence) for youth with particular mental health comorbidities, providing additional support with managing appointments and targeted strategies to assist with remembering appointments, planning for and physically getting to appointments and practicing scheduling before transfer to reduce some of the pressure of the first adult visit.

Barriers and facilitators to medication adherence have been well studied amongst individuals with multiple chronic conditions across the lifespan;^37^ however, this study expands our understanding of multimorbidity and medication adherence during pediatric–adult service transitions. Prior research demonstrates that adherence to prescribed medications in older adults with multimorbidity is suboptimal, possibly due to the complexity of their treatment regimens, the number of medications and the costs of filling prescriptions.^37^ Youth in the current study did not cite financial barriers or the number of medications as impacting their adherence to treatment. They did, however, describe challenges with managing their medications while experiencing mental health symptoms, including forgetfulness and disruptions to daily routines. Our results demonstrate the utility of care providers inviting youth to expand on their medication management strategies in clinical encounters and encouraging them to reflect on how their mental health affects these practices, especially when taking on these roles for the first time in preparation for the adult healthcare system.

The ability to self‐advocate through communicating one's needs to healthcare providers is a core component of successful chronic condition self‐management.^42^ In fact, evidence suggests that individuals with health or mental health conditions who openly communicate with healthcare providers and express their treatment preferences obtain more information to support decision making and experience fewer symptoms.^43^, ^44^, ^45^, ^46^ While our phase 1 results demonstrated youth with co‐occurring conditions were confident in talking with providers, there was dissonance between the phase 1 and phase 2 findings surrounding this domain. Some youth felt greater courage and insight into articulating their emotional needs as a result of engaging with mental health providers; however, the qualitative findings also revealed that the presence of a mental health comorbidity made communicating one's needs independently in adult care difficult in certain instances. For instance, phase 2 participants described feeling immobilized, fearful of responding incorrectly to questions and lacking the organizational skills to take in large amounts of information independently when severe anxiety or ADHD symptoms were present. This speaks to the challenges of applying median TRAQ scores to individual youth with unique experiences, coping strategies and mental health symptoms. It also underscores the inherent limitations of self‐reported surveys like the TRAQ, including social desirability bias.^47^

The phase 2 results are largely consistent with prior research in the adult population which identified that feelings of hopelessness, high levels of emotional distress and fears of wasting healthcare providers' time were barriers to effective communication and self‐advocacy in patient–provider interactions.^48^, ^49^ The present study expands on the literature in this field by focusing specifically on the impact of co‐occurring physical and mental health symptoms on one's ability to ask questions, respond to provider questions and articulate one's needs while adapting to newfound responsibility in the adult healthcare system. Phase 2 findings demonstrated that while extensive experience interacting with different service providers may enhance feelings of preparedness to communicate with care teams, mental health symptoms also contributed to feelings of pressure, worry about wasting providers' time or not having the appropriate responses to provider questions.

The progression of self‐management, communication and advocacy skills in youth transitioning to adult care is dependent upon a series of interwoven factors ranging from one's mental health symptoms, familial support, the complexity of their chronic condition(s) and their relationships with care providers, all of which are endorsed in the social‐ecological model of adolescent and young adult readiness for transition.^8^ This study's integrated insights underscore the limitations associated with self‐reported surveys administered at singular time points to measure complex concepts, like transition readiness. Fluctuating symptoms, for instance, cannot be captured within a cross‐sectional survey. Indeed, inherent difficulties exist with using an average transition readiness assessment score to condense the totality of a youth's experience, including sociodemographic background, relationships, beliefs, culture and community connectedness.^8^ Our findings demonstrate that tools like the TRAQ should be used cautiously to guide clinical care, given transition plans should be personalized according to need. This study highlights the benefits of using qualitative and mixed methods approaches to elicit nuanced understandings of how preparedness for adult care is shaped by the presence of mental health comorbidities. Further research using multiple data sources is needed to develop transition readiness tools and person‐centered interventions for youth with co‐occurring health and mental health conditions.

### 4.1 Limitations

These findings should be interpreted in light of several limitations. First, selection bias^50^ may have occurred in both phases 1 and 2 of this research. In phase 1, the data were obtained through a randomized controlled trial and did not represent a random sample of the general population. It is possible that the presence of severe mental health symptoms at the time of study recruitment (e.g., active psychosis or suicidality), for instance, precluded some youth from enroling in the trial. In Phase 2, those who agreed to participate in a qualitative interview may have been more organized and/or motivated than those in the phase 1 sample, contributing to possible differences in readiness across the two cohorts. Regarding the qualitative sample, it is possible that the experiences of youth with differing diagnoses, cultural and/or socioeconomic backgrounds could have strengthened this research by contributing new perspectives on this topic. It should be noted, however, that a demographically and clinically diverse sample of youth was obtained, and that the richness of the qualitative data^30^ guided the decision to cease recruitment efforts.

There was slight variation in the age ranges of participants in phases 1 and 2 of this research and the qualitative participants were not recruited from the quantitative sample which could contribute to inconsistencies between the quantitative and qualitative results. Phase 1 participants were between ages 16–18 (pretransfer) given the eligibility criteria for the TNT and the fact that baseline TRAQ scores (which were administered at the time of enrolment) were used in the descriptive analysis. This group's TRAQ scores, therefore, measured perceived and not actual levels of readiness given they had not yet transferred to adult care. Phase 2 participants ranged from 16 to 20 years old. It is possible the older participants who had more time to adapt to the adult system contributed to this sample appearing more prepared than those in phase 1; however, older age has been associated with greater readiness as measured by the TRAQ in the literature.^51^, ^52^ Though youth did reflect back on their levels of preparedness (as opposed to current readiness), which partly addressed this limitation, this may have been challenging for those with memory issues within our sample.

While youth in phases 1 and 2 self‐reported their mental health diagnoses, they were not asked to define the prominence, degree or severity of their symptoms, nor their coping strategies for managing these symptoms. Given the potentially important roles of symptom severity and coping in transition readiness,^53^ future research should continue to investigate these concepts using a combination of quantitative and qualitative data. Finally, the version of the TRAQ used in this study was developed in the United States before the COVID‐19 pandemic. As such, certain questions (e.g., regarding insurance) were less relevant to youth in Canada where this study took place. The impact of COVID‐19 on transition readiness and the refinement of existing tools to capture the nuances of virtual service delivery, for instance, should be considered in future research.

## 5 CONCLUSION

This patient‐oriented, mixed methods study yielded novel insights about how youth with co‐occurring conditions develop competencies related to self‐care, self‐advocacy and self‐management in preparation for service transitions. It illuminated the impact of mental health comorbidities on transition readiness in youth with chronic conditions using TRAQ subscale scores and qualitative interviews. Our results suggest that while instruments like the TRAQ may be useful for examining a group's average transition readiness (e.g., in assessing an intervention designed to improve transition readiness), they should not be used to guide individual decisions in the absence of in‐depth conversations with youth. Individualized transition plans should be developed in partnership with youth to address their goals, mental health symptoms, coping strategies and available support.

## AUTHOR CONTRIBUTIONS

Brooke Allemang conceptualized the study, collected and analyzed the data and drafted the manuscript. Susan Samuel and Gina Dimitropoulos substantially revised the manuscript and provided mentorship and supervision in study conceptualization, research design, data analysis and manuscript preparation. Katelyn Greer, Keighley Schofield, Karina Pintson, Megan Patton and Marcela Farias substantially revised the manuscript and conducted data analysis and interpretation. Kathleen C. Sitter, Scott B. Patten and Andrew S. Mackie substantially revised the manuscript and provided mentorship throughout study conceptualization and manuscript preparation. All authors read and approved the final manuscript.

## CONFLICT OF INTEREST STATEMENT

The authors declare no conflict of interest.

## ETHICS STATEMENT

Ethical approval for this research was obtained from the University of Calgary Conjoint Health Research Ethics Board. Written informed consent was obtained for individuals enroled in the Transition Navigator Trial (phase 1) and for individuals who participated in qualitative interviews (phase 2).

## Supporting information

Supporting information.

## Data Availability

The data that support the findings of this study are available on request from the corresponding author. The data are not publicly available due to privacy or ethical restrictions.
