# Supplementary material for: Transition readiness of youth with co‐occurring chronic health and mental health conditions: A mixed methods study
Source: Health Expect. 2023 Jul 14;26(6):2228–44. doi: 10.1111/hex.13821 (PMC10632650; doi:10.1111/hex.13821)
Supplement: Supplementary file 1 — Supporting information. [file HEX-26--s001.docx]

**Good Reporting of A Mixed Methods Study (GRAMMS) Checklist**^26^

| **Guideline** | **Section: Page** |
| --- | --- |
| Justification to use a mixed methods approach to the research question | Methods; Study Design  Pages 6-7 |
| Articulation of the design in terms of purpose, priority, and sequence of methods | Methods; Study Design  Pages 6-7 |
| Describe each method in terms of sampling, data collection and analysis | Methods  Pages 8-11 |
| Delineate where and how integration occurs and who has participated in it | Methods  Page 10-11 |
| Describe any limitation of one method associated with the presence of another | Methods & Results  Pages 6, 10-12 |
| Describe insights gained from mixing or integrating methods | Results; Mixed Methods Insights  Pages 12-18 |
